# Evaluation of a cognitive affective model of physical activity behavior

**DOI:** 10.15171/hpp.2020.14

**Published:** 2020-01-28

**Authors:** Paul D. Loprinzi, Sara Pazirei, Gina Robinson, Briahna Dickerson, Meghan Edwards, Ryan E. Rhodes

**Affiliations:** ^1^Exercise & Memory Laboratory, Department of Health, Exercise Science and Recreation Management University of Mississippi, Oxford, MS, USA; ^2^Behavioural Medicine Laboratory, School of Exercise Science, Physical and Health Education, The University of Victoria, Victoria, BC, Canada

**Keywords:** Cognition, Awareness, Metacognition, Mental processes

## Abstract

**Background:** To empirically evaluate a cognitive affective model of physical activity. This bidirectional, cyclical model hypotheses that executive control processes directly influence habitual engagement in exercise and also directly subserve the exercise-induced affective response to acute exercise associated with future physical activity.

**Methods:** The present study employed a one-week prospective, multi-site design. Participant recruitment and data collection occurred at two separate University sites (one in the United States and the other in Canada). Participants completed a bout of treadmill exercise, with affect and arousal assessed before, during and after the bout of exercise. Subjective and objective measures of executive function were assessed during this visit. Following this laboratory visit, seven days of accelerometry were employed to measure habitual engagement in physical activity.

**Results:** Within our inactive, young adult sample, we observed some evidence of 1) aspects of executive function were associated with more light-intensity physical activity in the future (1-week later) (r = 0.36, 95% CI = -0.03 to 0.66, *P* = 0.07), 2) aspects of executive function were associated with post-exercise affect (r = -0.39, 95% CI = -0.67 to -0.03, P = 0.03) and forecasted affect (r =0.47, 95% CI = 0.11 to 0.72, *P* = 0.01), and 3) aspects of acute exercise arousal and affect were associated with current mild-intensity physical activity behavior (r = 0.41, 95% CI = 0.04 to 0.68, *P* = 0.03).

**Conclusion** : We demonstrate partial support of a cognitive-affective model of physical activity.

## Introduction

Regular participation in physical activity is associated with reduced risk of numerous cardiovascular and psychological diseases.^1^ Physical inactivity, on the other hand, is responsible for substantial economic burden.^2^ Thus, physical activity promotion is of critical importance for individual and societal health.

In order to effectively promote physical activity, it is necessary to understand the antecedents of this complex behavior. Physical activity is influenced by a multitude of factors, ranging from individual-level beliefs to societal-level factors.^3^ While acknowledging this complexity, the purpose of this present study was to empirically evaluate a cognitive affective model of physical activity.

Research demonstrates that habitual engagement in physical activity alters brain structure and function,^4^ and this, in turn, may facilitate future exercise behavior.^5^ As such, the physical activity-brain relationship is thought to occur bi-directionally.^6^ A notable cognitive outcome that is favorably influenced by physical activity is executive function. This cognitive outcome is often operationalized as a higher-order cognition, governed by the prefrontal cortex, which is responsible for the engagement in goal-directed behaviors and inhibition of goal-inconsistent behaviors.^7^ Executive function has been shown to moderate the intention-behavior relationship for several behaviors, including physical activity.^8^

In addition to physical activity behavior being directly regulated by executive control processes,^5^ executive function may indirectly influence physical activity via shaping affective-related processes. As an example, physical activity-induced affective response,^9^ which is associated with future physical activity behavior,^10^ may be shaped from an individual’s cognitive interpretations of the physiological experience. The physical activity-related affective responses may, hypothetically, even occur independent of executive control-related processes, via bottom-up, automatic processes.^11^

**Figure 1:**
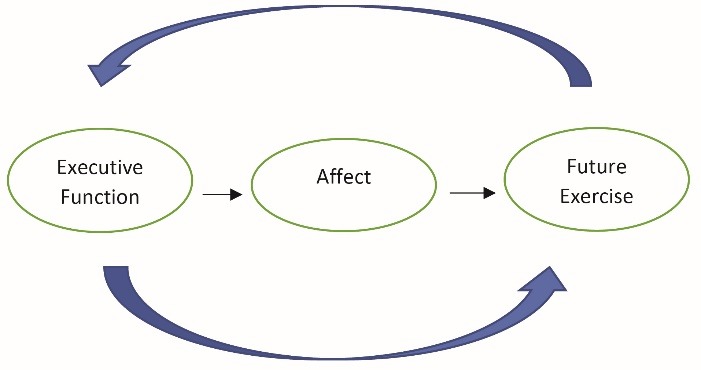
Hypothesize model of the interrelationships between executive function, affect, and future exercise behavior.

Couched within the above, executive function may directly and indirectly (via affective responses) influence physical activity behavior. We previously developed and discussed this model in detail.^12^ The purpose of this study, written as a brief report, was to empirically evaluate this conceptual model (Figure 1) within an inactive population. We hypothesized that executive function would be positively associated with both acute exercise-induced affect and habitual physical activity behavior. Further, we hypothesized that the exercise-induced affective response would be positively associated with habitual physical activity behavior.

## Materials and Methods

### Study design

The present study employed a one-week prospective, multi-site design. Participant recruitment and data collection occurred at the University of Mississippi (USA) and University of Victoria (Canada). Participants were recruited using a non-random, convenience-based sampling approach.

### Procedures

Participants completed one main laboratory visit. This visit involved anthropometric assessments (measured height and weight), completing several surveys (demographic, habitual physical activity), several cognitive function tasks, and a treadmill bout of acute exercise with an assessment of their affective response to the acute bout of exercise. At the end of the visit, participants were given a waist-mounted accelerometer and were asked to wear it for the 7 subsequent days. Participants arrived back at the laboratory 1-week later to return the accelerometer.

### Participants

The sample included 32 participants. Participants were young healthy adults between 18 and 45 years of age. Further, participants were eligible for participation if they did not have a walking disability; answered “no” to all 7 questions on the Physical Activity Readiness Questionnaire; were not taking any medications known to influence cognition or emotion; did not have a concussion or head injury within 30 days prior to participation; were not pregnant; did not have a diagnosis of attention deficit hyperactivity disorder; did not take marijuana or other drugs known to influence emotion or cognition within 7 days prior to participation; were not a smoker; were not a regular alcohol drinker (> 30 drinks/month for women; >60 drinks/month for men); did not have a strong aversion to treadmill exercise (≥ 8 on a 0-10 aversion scale); and did not -report meeting physical activity guidelines (≥ 150 min/week of moderate-to-vigorous physical activity or ≥75 min/wk of vigorous physical activity).

### Measures

*Executive function*

Four measures of executive function were employed, including tasks measuring planning, working memory, inhibitory control and task switching. Three of these tasks (planning, working memory, inhibitory control) were objectively assessed using computer-based software (Inquisit software). Planning-based cognition was assessed using the Tower of London task^13^; working memory was assessed from the Operation Span (OSPAN) task^14^; and inhibitory control was measured from the Stroop word-color task.^15^ Lastly, the task switching measure involved subject perceptions to two switching tasks.

The Tower of London task involves trying to rearrange balls on a peg board to create a given pattern. Twelve trials were employed, with the total score calculated as the sum of the scores from each trial. A higher score is indicative of better planning-based cognition. The OSPAN task involved the presentation of visual sequences of letters ranging from 3-7 letters that were to be recalled at the end. Each letter in the sequence was preceded by a simple arithmetic problem followed by a proposed solution and participants had to decide whether the proposed solution was correct. Five outcome metrics were calculated for the OSPAN, including the absolute score (sum of all perfectly recalled sets), total correct score (total number of letters recalled in the correct position), math total errors (total number of errors), speed errors (number of times they ran out of time in attempting to solve a problem), and math accuracy errors (number of times they solved the operation incorrectly). The Stroop word-color task involved color words (e.g., “red”) written in a color and participants were asked to indicate the color of the word (not its meaning) by specific key presses. There are 84 total trials, consisting of 4 colors (red, green, blue, black) x 3 color-stem congruency (congruent, incongruent, control) x 7 repetitions. The congruent trials involved the color word and the color it presented being the same; incongruent trials involved the color word being different than the color it was presented in (e.g., it read GREEN, but this word was not in the green color); and the control trials involved colored rectangles. The outcome measure is the average latency (in milliseconds [ms]) of the correctly identified congruent, incongruent and control trials.

Task switching was evaluated from -perceptions on the performance of two switching tasks, developed for this project. First, participants were asked, within a 60-sec period, to “*write as many numbers as you can in ascending order, starting with the number 2 and counting by 7* .” After this task, participants were asked, within a 60-second period, to “*write as many girl names as you can think of that start with the first half of the alphabet only (A-M)* .” After completing this second task, they were asked four separate questions:

- “*How focused did you feel during both tasks* ?”
- Response options ranged from 1 (not focused at all) to 100 (completely focused)
- “*How easy was it to switch from one task to the other* ?”
- Response options ranged from 1 (extremely difficulty) to 100 (extremely easy)
- “*How creative did you feel your responses were for the second task* ?”
- Response options ranged from 1 (not creative at all) to 100 (very creative)
- “*how easy was it to avoid writing boy names and names that started with letters in the second half of the alphabet* ?”
- Response options ranged from 1 (extremely difficulty) to 100 (extremely easy)

*Acute treadmill exercise with affect assessment*

Participantswalked on a treadmill for 15 minutes. The specific instructions given to participants were, “*Please walk for 15 minutes on this treadmill at a brisk walking pace; a pace you would walk if you were late for catching the bus; thus, this should not be a leisurely walk* .” Half-way through the 15-min bout of exercise, participants completed the Feeling Scale (FS) and Felt Arousal Scale (FAS) via paper-and-pencil (details described elsewhere^9^); they were asked to circle their answers with the scales on a clipboard. Prior to and immediately following the bout of exercise, participants also completed the FA, FAS, and Affective Circumplex Scale (ACS) (to measure distinct affect parameters) via pen and paper. These assessment periods (before, midpoint of exercise, and immediately post-exercise) were selected based on prior research utilizing these temporal periods that showed positive affective responses from an acute bout of exercise.^9^ Following this, participants completed a scale assessing forecasted exercise pleasure.

The FS asked participants to indicate the most appropriate number to represent how they felt in the current moment, ranging from -5 (very bad) to +5 (very good). The FAS asked participants to indicate their level of arousal, ranging from 1 (low arousal) to 5 (high arousal). The ACS asked participants to indicate how much, at the present moment, they felt the following emotions (ranging from 0-100), happy/cheerful, excited energetic, content, sad, angry, anxious/worried, tense/wound-up, and fatigued. The forecasted pleasure item, ranging from -100 (most unpleasant imaginable) to 100 (most pleasant imaginable), asked the following, “*If you repeated the exercise session again in the future (e.g., several months from now), how do you think it would make you feel* ?”

### Habitual physical activity

Physical activity was assessed subjectively and objectively. Participants completed the Physical Activity Vital Signs questionnaire,^16^ which involves two items, “*On average, how many days per week do you engage in moderate to strenuous exercise* ?” and “*On average, how many minutes do you engage in exercise at this level* ?” Based on the product of these two items, we calculated the amount of time spent per week in moderate to vigorous physical activity (MVPA). self-reported mild physical activity was assessed from the Godin Leisure-time Questionnaire.^17^

Physical activity was objectively assessed using a GT3x ActiGraph accelerometer. The accelerometer was worn on an elastic belt over the hip for 7 consecutive days. A valid day of accelerometer monitoring included at least 600 minutes of wear time, with nonwear defined as a period of 60 consecutive minutes of zero activity counts, with the allowance of 1-2 minutes of activity counts between 0 and 100.^18^ Estimates of light, moderate, and vigorous-intensity physical activity were determined from the Freedson cut-points.^19^

### Statistical analyses

All analyses were computed in JASP (v. 0.10). Bivariate zero-order correlations were computed among the evaluated constructs. In alignment with the evaluated model, correlation analyses were conducted to evaluate the association between executive function and physical activity, executive function on affect, and affect on physical activity. Statistical significance was established at 0.05.

## Results

### Demographic and behavioral characteristics

Table 1 displays the demographic characteristics of the sample. Participants, on average, were 21.1 years of age, with the sample being predominately female (81.3%) and non-Hispanic black (62.5%). Participants had an average body mass index in the overweight range (27.0 kg/m^2^).

Table 2 displays the physical activity estimates of the sample. Participants self-reported engaging in 32.3 (38.7) min/week of MVPA. Regarding the accelerometer-derived estimates, the average wear time per day was 843.5 (215.3) min/d. Participants had an average of 3.8 (2.0) valid accelerometer days (i.e., ≥ 600 min/d of wear time). The proportion of wear time per day spent in sedentary behavior was 73.0% (8.2), 21.8% (7.5) for light, 5.1% (2.4) for moderate, 0.001% (0.001) for vigorous, and 0.0002% (0.001) for very vigorous physical activity.

### Cognitive Function Characteristics

Table 3 displays the cognitive function characteristics of the sample.

### Physiological (heart rate) and affective response from acute exercise

Table 4 displays the physiological and affective responses from the acute bout of treadmill exercise.

### Model evaluation

#### Executive Function → Affect

Post-exercise tension was inversely associated with the task switching item #4 (r = -0.39, 95% CI = -0.67 to -0.03, *P* = 0.03). That is, those who perceived they did better on the cognitive task had less tension after the acute bout of exercise. Task switching performance on item 4 was positively associated with forecasted pleasure (r = 0.47, 95% CI = 0.11 to 0.72, *P* = 0.01). That is, those who perceived they did better on the cognitive task had a greater forecasted pleasure. Task switching item #3 was positively associated with forecasted pleasure (r = 0.37, 95% CI = 0.00 to 0.66, *P* = 0.05). That is, those that thought they were more creative in the cognitive task had a higher forecasted pleasure in PA.

#### Affect → Physical Activity

Post-exercise fatigue was inversely associated with self-reported MVPA (r = -0.38, 95% CI = -0.66 to -0.01, *P* = 0.04). That is, those with greater fatigue after the acute bout of exercise engaged in less self-reported MVPA. Post-exercise FAS was inversely associated with accelerometer-derived very vigorous-intensity physical activity (r = -0.46, 95% CI = -0.74 to -0.06, *P* = 0.02). That is, those with a higher perceived arousal score after the acute bout of exercise engaged in less habitual vigorous exercise. Lastly, FS during the acute bout of exercise was positively associated with weekly engagement in mild-intensity physical activity (r = 0.41, 95% CI = 0.04 to 0.68, *P* = 0.03).

#### Executive Function → Physical Activity

Task switching item #1 was positively associated with accelerometer-derived light PA (r = 0.36, 95% CI = -0.03 to 0.66, *P* = 0.07). That is, those that had a higher perceived level of focus during the cognitive tasks engaged in more light-intensity PA.

## Discussion

We previously developed and discussed an integrated, cognitive-affective model of physical activity.^12^ This bi-directional, cyclical model hypotheses that executive control processes directly influence habitual engagement in exercise and also directly subserve the exercise-induced affective response to acute exercise associated with future physical activity. In the present study, we provide some empirical support for this model. That is, within our inactive, young adult sample, we observed some suggestive evidence of (1) aspects of executive function were associated with more light-intensity physical activity in the future (1-week later), (2) aspects of executive function were associated with post-exercise affect and forecasted affect, and (3) aspects of acute exercise arousal and affect were associated with current mild-intensity physical activity behavior and future physical activity levels. This latter finding has important public health implications as mild-intensity physical activity is associated with favorable health outcomes and is a physical activity intensity level that the broader population is more likely to adopt.^20,21^

In our previous hypothesis paper,^12^ we detailed prior research that provides support for each of the delineating pathways within our model. As such, the present findings align with prior work and support our hypothesized model. As an example, we have previously shown that executive function is associated with less sedentary behavior in the future.^22^ Less empirical work has specifically evaluated the association between executive function and the affective response to exercise. Such a relationship, however, is plausible, as the affective response to exercise may arise from cognitive interpretations of physiological and psychological experience.^23^ Our present study provides some suggestive support for these assertions, as we demonstrated that greater perceptual performance on a cognitive task was associated with more favorable post-exercise affect and forecasted affect. Lastly, in alignment with the broader literature,^10^ the results from our present study demonstrate that the affective response during an acute bout of exercise was positively associated with current and future physical activity behavior. Notably, we did not observe a negative affective response during the bout of exercise, which one might expect, particularly in an inactive sample. This is likely attributed to the minimal MVPA engagement of the sample as well as the utilization of a -paced (below critical intensity that initiates an undesirable affect response) exercise session.^24^

Limitations of this study include the small, homogenous sample. As such, it was not possible to appropriately control for potential confounding factors. Although the intent of the present study was to provide an initial evaluation of our hypothesized model, future studies should continue to evaluate this model using a larger, more representative sample. Such an approach would likely induce more reliable estimates. Given our limited sample, and potential risk of committing a type 2 research error, we intentionally chose not to adjust for multiple comparisons.^25^ If doing so, several of the statistically significant associations observed in the present study would no longer be statistically significant. As such, our findings should be interpreted accordingly. Relatedly, and although several associations were statistically significant, many were not. Thus, future research is needed to help better understand these complex, interrelationships. Despite these limitations, strengths of this study include its novelty, objective and subjective assessments of physical activity and cognition, comprehensive assessment of these parameters, experimental induction of acute exercise, one-week prospective evaluation of physical activity, and utilization of a relatively inactive sample.

## Conclusion

In conclusion, in this preliminary evaluation, we demonstrate partial support of a cognitive-affective model of physical activity. That is, aspects of executive function were directly associated with physical activity. Further, executive function was associated with exercise-induced affect, which in turn, was associated with future physical activity behavior. If confirmed by future research, then these findings will underscore the importance of shaping both cognitive and affective aspects to optimize physical activity promotion.

## Ethical approval

This study was approved by the ethics committee at both institutions. Participant consent was obtained prior to data collection.

## Competing interests

The authors declare that they have no competing interests.

## Funding

No funding was used to prepare this manuscript.

## Authors’ contributions

PDL and RER conceptualized and supervised the study and drafted the manuscript. Authors SP, GR, BD, and ME collected data.

## Acknowledgments

We also acknowledge the hard work of ethics application organization and questionnaire formatting and preparation by Stina Grant and Christina McLean.

**Table 1:** Demographic characteristics of the sample

**Variables**	**Point Estimate**	**Standard Deviation**
Age, mean years	21.1	1.2
Gender, % female (n = 26)	81.3	
Race, %		
Non-Hispanic white (n=8)	25.0	
Non-Hispanic black (n=20)	62.5	
Other (n=4)	12.5	
BMI, mean kg/m^2^	27.0	7.4

Abbreviation: BMI, body mass index.

**Table 2:** Physical activity estimates of the sample

**Variables**	**Point Estimate**	**Standard Deviation**
Selfself-reported		
MVPA, mean min/wk	32.3	38.7
Objectively-measured		
Sedentary, mean min/d	612.0	147.5
Light, mean min/d	186.1	93.0
Moderate, mean min/d	42.5	21.5
Vigorous, mean min/d	0.75	1.4
Very vigorous, mean min/d	0.16	0.74

Abbreviation: MVPA, moderate to vigorous physical activity.

**Table 3:** Cognitive outcomes of the sample

**Variables**	**Point Estimate**	**SD**
Objective Measures		
Tower of London, mean	30.2	3.4
OSPAN, mean		
Absolute score	33.9	17.4
Total correct score	51.4	17.9
Math total errors	5.4	6.4
Speed errors	1.1	1.1
Math accuracy errors	4.3	5.7
Stroop, mean (milliseconds) reaction time		
Congruent	1125.0	372.8
Incongruent	1374.0	458.9
Control	1105.0	354.5
Subjective Measures		
How focused did you feel during both tasks?	74.7	28.2
How easy was it to switch from one task to the other?	77.4	25.2
How creative did you feel your responses were for the second task?	52.4	32.1
How easy was it to avoid writing boy names and names that started with letters in the second half of the alphabet?	70.6	29.6

Abbreviation: SD, standard deviation.

**Table 4:** Physiological (heart rate), perceptual (RPE) and affective responses to the acute treadmill exercise

**Variables**	**Point Estimate**	**Standard Deviation**
**Heart rate**		
Rest	72.1	10.9
Midpoint	105.8	18.0
Endpoint	106.9	25.6
Post	80.6	16.7
**RPE**		
Rest	7.1	1.7
Midpoint	10.0	1.5
Endpoint	10.7	1.4
Post	7.2	1.8
**FS**		
Rest	2.58	1.4
Midpoint	2.90	1.2
Post	2.89	1.2
**FAS**		
Rest	2.58	1.0
Midpoint	3.43	0.8
Post	3.29	1.0
**ACS**		
Happy		
Pre	57.5	24.4
Post	65.1	23.1
Excited		
Pre	49.2	27.6
Post	60.9	23.9
Content		
Pre	76.5	23.6
Post	71.7	25.6
Sad		
Pre	11.2	20.9
Post	7.2	14.4
Angry		
Pre	3.6	8.5
Post	7.4	18.2
Anxious		
Pre	17.8	26.7
Post	13.8	21.6
Tense		
Pre	17.6	31.2
Post	14.0	21.9
Fatigue		
Pre	21.1	29.3
Post	18.0	21.4

Abbreviations: FAS, Felt Arousal Scale; FS, Feeling Scale; ACS, Affective Circumplex Scale; RPE, rating of perceived exertion.
